# Lasting effects of brief health equity/implicit bias education for academic clinicians: From learning to action 

**DOI:** 10.12688/mep.20799.1

**Published:** 2025-07-11

**Authors:** Janice Sabin, Grace Guenther, Kris Piu Kwan Ma, Bernadette York, Wendy Barrington, Bianca Frogner

**Affiliations:** 1 University of Washington School of Medicine, Seattle, Washington, USA; 2 University of Washington School of Nursing, Seattle, Washington, USA

**Keywords:** implicit bias education, lasting effects, implicit and explicit bias

## Abstract

**Purpose:**

The purpose of this study was to determine whether there were lasting effects of brief implicit bias education on the teaching and practice of academic clinicians one year after taking the course.

**Method:**

This was a multi-method study. We followed up with a sample of 119 academic clinicians who completed the baseline study December 2019. Recruitment for the current study was conducted between December 2020 and March 2021. Participants responded online to survey questions about whether the course had an impact on their teaching and practice. We categorized qualitative responses to these questions using Prochaska & DiClemente's Stages of Change Model of Behavior Change. Implicit and explicit race and gender bias data were collected at baseline.

**Results:**

Response rate was 48.7% (N=56). Participants were 75.0% female, 66.1% White, and 67.9% were MDs. We found moderate implicit bias favoring White people (Cohen’s d= 0.60), and strong implicit gender bias associating males rather than females with the concept of “career” (Cohen’s d= 0.95). One year after taking the course, 62.5% of participants reported that the content of the course had an impact on their teaching and 41.4% reported the course had an impact on their clinical practice. Across all open-ended questions, 63.8% participants reported having taken at least one action (actual behavior change) in teaching and/or practice due to the course.

**Conclusions:**

There were lasting effects of implicit bias education on participants’ teaching and practice. Brief implicit bias education moved clinicians toward taking action to improve their teaching and practice.

## Introduction

Healthcare professionals’ implicit bias is one of many factors contributing to disparities in healthcare and health outcomes ^1, 2^. Implicit bias is defined as “attitudes or stereotypes that affect our understanding, decision-making and behavior without our even realizing it” ^3^. Similar to the general population, implicit bias exists among health professionals in the areas of race, gender, ethnicity, sexual orientation, age, weight, mental illness, and other areas ^1, 4– 6^. Healthcare professionals’ implicit bias contributes to poor clinician-patient communication, disparities in pain management, prescribing lipid-lowering medication for women, treatment of coronary heart disease, and other areas ^1, 7, 8^.

Implicit bias education for students, trainees and clinicians in practice has been integrated into some medical school curricula and healthcare system clinician trainings, but not all. There is no clear evidence of optimal implicit bias curricula content, or educational evaluation strategies ^9^. The focus of most implicit bias education is not to eliminate the bias that is by its nature ubiquitous, hidden, and has proven to persist in the long term ^10^, but rather, to recognize how bias manifests and manage the impact of implicit attitudes and beliefs in teaching, practice and healthcare delivery ^11, 12^. At the University of Washington, authors of this study found that academic clinical faculty needed additional foundational health equity/implicit bias education to feel competent in teaching about implicit bias in healthcare. In 2017, we developed a brief, online course for academic clinicians titled, *Implicit Bias in the Clinical and Learning Environment,* to meet this need.

Evaluation of health equity/implicit bias education for clinical faculty who teach is in its early stages. Little is known about lasting effects of implicit bias education on clinicians’ behavior. Studies have found that brief online implicit bias education can increase bias awareness and intentions to change behavior ^13– 15^. In this study, we returned to a sample of primary care clinical faculty one year after they took a brief online course and used the method of personal reflection for participants to report on the impact of the course in their teaching and clinical practice over the past year. The aim of this study was to explore whether and how the course had an impact on clinicians’ teaching and practice during the one year following taking the course. Our research questions were 1. *Does health equity/implicit bias education have lasting effects on teaching and practice, and if so, how?* and *2. Is clinician implicit and explicit bias associated with lasting effects of health equity/implicit bias education?*

## Methods

### Study design and sample

This multi-method study returned to a sample of academic primary care clinicians who completed a survey and online health equity/implicit bias education, *Implicit Bias in the Clinical and Learning Environment* ^14^, between September 2019 and December 2019, which we refer to as our baseline study, publicly available at [ https://depts.washington.edu/somalt/implicitbias-pi/story.html]. All participants who completed the baseline study were invited to participate in the current follow up study to evaluate lasting effects of the course. The follow up study was conducted between December 2020 and March 2021. Our baseline sample consisted of 119 U.S. academic family, internal, and emergency medicine providers, nurse practitioners, and physician assistants recruited from all nine U.S. Census Divisions ^14^. Demographic and background information was collected in the baseline study, including information on personal and professional characteristics, and implicit and explicit race and gender bias. In the follow up survey, we updated participants’ work position and asked four reflection questions about if and how the course impacted their teaching and clinical practice over the past year. The University of Washington Human Subjects Institutional Review Board approved the study as minimal risk [Baseline study approval IRB # 00006978, Modification for follow up IRB #00008382, approved 11/13/2020].

**Implicit Bias Education:** Participants engaged in brief online health equity/implicit bias education at time of the study baseline. The course was developed by a team (including author JS), with expertise in medical education, adult teaching and learning theory, social determinants of health, implicit bias in healthcare, medical school clinician-administrators and academic clinicians who practice across a wide range of settings to serve as foundational information for academic clinicians who teach. The course was designed to be brief so that it would not over burden busy clinicians. The course had three learning objectives: 1) define implicit bias and how it is manifested in health care, 2) recognize how implicit bias may be operating in the clinical setting and learning environment, and 3) apply strategies that can be used to minimize impact of implicit bias. Although the course was developed prior to publication of Sukhera & Watling’s (2018) implicit bias recognition and management (IBRM) framework for implicit bias education, the course incorporated many of the framework’s features such as creating a safe environment, content on the science of implicit bias and increasing awareness of implicit bias ^16^. Course content included: the history of racism in medicine, social determinants of health, evidence of discrimination in healthcare, the science of implicit bias, evidence about how implicit bias manifests in clinical care and the learning environment, and numerous strategies to mitigate the impact of implicit bias on teaching and practice. Upon entering this follow up study, participants had the opportunity to revisit the course.

**Implicit Bias Measures:** In the baseline study we measured participant implicit race and gender bias using the standard Race Implicit Association Test (IAT) and Gender-Career IATs [available at: https://implicit.harvard.edu/implicit/] designed by scientists at Project Implicit using best practices for IAT design ^17, 18^. The IAT is a widely used, computer-based test of implicit social cognition that measures the relative strength between positive and negative associations toward one social group compared with another ^13^. The Black-White Race IAT asks test takers to sort and pair facial images of the target concept of race (faces of Black People and faces of White People) and words that represent “good” (e.g., glorious) or “bad” (e.g., yucky) as they appear on a computer screen. The Gender-Career IAT measures gender stereotypes using the target concepts of “male” (represented by traditionally male and female names, (e.g., Ben) versus “female” (e.g., Rebecca) and the concept of “career” represented by words associated with career (e.g., office) versus “family” represented by words associated with family (e.g., home). The difference in time taken to sort and pair these images and words as they rapidly appear on computer screen, measured in milliseconds, demonstrates the strength of their automatic association ^19^. The IAT was used only as a measure of bias and was not used as an intervention to increase awareness of participants’ personal bias. To explore the topic further, participants were given the Project Implicit web address [ https://implicit.harvard.edu/implicit/] which provides the public with an opportunity to take IATs with personal feedback.

**Explicit Bias Measures:** At baseline, we used standard explicit measures that correspond with the Race IAT [available at: https://implicit.harvard.edu/implicit/] to measure respondents’ preference for Black People versus White People on a 7-point preference response scale ^20^. The explicit race measure question was: Which statement best describes you? *1*. *I strongly prefer Black People compared to White People; 2. I moderately prefer Black People compared to White People; 3. I slightly prefer Black People compared to White People; 4. I like Black People and White People equally; 5. I slightly prefer White People compared to Black People; 6. I moderately prefer White People compared to Black People; and 7. I strongly prefer White People compared to Black People*. For the Gender IAT, we used two separate explicit measures: one that asked about association of gender (male versus female) with career only, and one that asked about association of gender with “career” versus “family” only, using the same format and 7-point scale.

**Open-ended, Reflective Questions:** Participants were asked four yes/no reflective questions and to provide a typed response to the following prompts: *1. Reflecting on this course, has the content of the course impacted your teaching and/or mentoring? If yes, how? 2. Has the content of the course impacted your teaching and/or mentoring due to the COVID-19 pandemic, the current social justice and equality movement, or the current healthcare policy debate? If yes, how? 3. Reflecting on this course, has the content of the course impacted your clinical practice? If yes, how? and 4. Has the content of the course impacted your clinical practice due to the COVID-19 pandemic, the current social justice and equality movement, or the current healthcare policy debate? If yes, how?* (Supplement 1).

**Quantitative Analysis:** Quantitative analysis consisted of descriptive statistics to characterize the sample and implicit and explicit bias scores and Pearson Product Moment correlation to assess associations between clinicians’ biases and impact of course. Similar to other studies using the IAT ^20^, we used Cohen’s d, a measure not affected by sample size, to assess IAT standardized effect size which can be interpreted as how different the result is from zero (no bias) ^21^. Cohen’s d is interpreted as follows: d = 0.2, small effect; d = 0.5, medium effect; and d = 0.80, large effect ^21^. Quantitative analysis was conducted using Stata (StataCorp. 2019. Stata Statistical Software: Release 16. College Station, TX: StataCorp LLC.) ^22^ and IBM SPSS Statistics for Macintosh (version 26.0, Armonk, NY: IBM Corp) ^23^. [see software availability statement]

**Qualitative Analysis:** We conducted a high-level thematic analysis of responses to the four reflective questions. We used the Prochaska & DiClemente Stages of Change Model of Behavior Change guide ^24^ which has previously been used to categorize qualitative data from bias interventions, as our analytical framework ^25^. The stages of change categories are; Precontemplation (no intention to change), Contemplation (considering change), Determination (preparing to change), Action (changed behavior), and Maintenance (maintaining new behavior) ^24^. First, study team members (JS, GG, KM, WB, BWY, BF) independently coded a subset of reflective question responses according to the Stages of Change model. The study team then collaboratively reviewed and finalized codes through an iterative process. Once codes were finalized, one team member (GG) applied the codes to the remaining questions and responses. Themes and sub-themes within each stage of the model were identified as a group. Qualitative analysis was conducted using Dedoose software (version 9.0.17) ^26^.

## Results

### Sample

Drawing from the original study sample of 119 participants, the follow up study response rate was 47.1% (N=56).

### Participants

Participants were 75.0% female, 66.1.0% White, and 67.9% were MDs ( Table 1). Compared to those who did not respond, this follow up study sample was more racially diverse (66.1% White vs. 81.0% White), younger (< age 50, 71.5% vs. 50.0%), more were MDs (67.9% vs. 46.6%), and more worked in an academic healthcare system (82.1% vs. 70.7%).

**Table 1.** Study Sample Characteristics (N=56).

	N (%)
**Gender**	
Male	14 (25.0%)
Female	42 (75.0%)
**Age**	
30–39	23 (41.1)
40–49	17 (30.4)
50–59	8 (14.3)
60+	8 (14.3)
**Ethnicity**	
Hispanic/Latino	4 (7.1)
Not Hispanic/Latino	52 (92.9)
**Race**	
Black/African American	9 (16.1)
Asian	7 (12.5)
White	37 (66.1)
Native Hawaiian/Other Pacific Islander	3 (5.4)
**Area of Practice**	
Medical Doctor (MD)	38 (67.9)
Nurse Practitioner (NP)/ Physician Assistant (PA)	9 (16.1)
Other	9 (16.1)
**System**	
Academic Healthcare System	46 (82.1)
Community Healthcare System	9 (16.1)
Other	1 (1.8)
**Region**	
Northeast	9 (16.1)
Midwest	14 (25.0)
South	22 (39.3)
West	11 (19.6)
	**M (SD)**
**Years in Clinical Practice**	16.0 (11.2)
**Years in Current Position**	7.85 (8.3)
**# Patients/Week Direct ** **Clinical Contact**	21.4 (10.6)

### Implicit and explicit measures

Our sample held moderate implicit race bias associating White People more readily with the concept of “good” than Black People (Cohen’s *d*= 0.60), and a strong implicit gender bias associating males rather than females with the concept of “career” versus “family” (Cohen’s *d*=0.94) ( Table 2). On explicit measures, we did not find evidence of explicit race bias (Cohen’s *d*= 0.14). We found a strong explicit association of “male” with “career” versus “family” (Cohen’s *d*= 0.71), and female with “family” versus “career” (Cohen’s *d*= 1.13).

**Table 2.** Implicit and Explicit Measures (N=56).

Implicit and Explicit Measures (a)	M	SD	P (b)	Cohen’s *d* (c)
**Implicit Race**	0.27	0.45	<0.001	0.60
**Implicit Gender**	0.33	0.31	<0.001	0.94
**Explicit Race**	0.11	0.76	0.29	0.14
**Explicit Career**	0.76	1.07	<0.001	0.71
**Explicit Family**	-1.07	0.95	<0.001	1.13

a) For implicit and explicit measures, a positive score favors White on Race IAT, male and career on the Gender IAT, and male with career on the explicit gender measure. A negative score favors Black on the Race IAT, female with career and female with family on the gender implicit and explicit measures. b) T-test, value=0 c) Implicit measures are a continuous measure ranging from -2.0, +2.0 with “0” signifying no bias. Explicit measures are on a scale that ranges from -3.0, +3.0, with “0” signifying no bias. d) Cohen’s *d* values are interpreted as follows: *d* = 0.2, small effect; *d* = 0.5, medium effect; and *d* = 0.80, large effect, Cohen, 1988

### Overall impact of the course

One year after taking the course, 35 participants (62.5%) reported that the content of the course had an impact on their teaching and 23 participants (41.4%) reported that the course had an impact on their clinical practice ( Table 3). Across all four open-ended questions, 63.8% participants reported having taken at least one action (actual behavior change) in teaching and/or practice due to the course.

**Table 3.** Impact of Course on Teaching and Practice and Stages of Change (N=56).

Question	Change due to Course	N (%)
1. Reflecting on this course, has the content of the course impacted your teaching and/or mentoring? If yes, How?		
	No	21 (37.5)
	Yes	35 (62.5)
	**If yes, Stages ** **of Change**	
	Contemplation	12 (25.0)
	Determination	2 (3.6)
	Action	17 (30.4)
	Maintenance	4 (7.1)
2. Has the content of the course impacted your teaching and/or mentoring due to the COVID-19 pandemic, the current social justice and equality movement, or the current healthcare policy debate? If yes, How?		
	No	30 (53.6)
	Yes	26 (46.4)
	**If yes, Stages ** **of Change**	
	Contemplation	8 (14.3)
	Determination	2 (3.6)
	Action	13 (23.2)
	Maintenance	3 (5.4)
3. Reflecting on this course, has the content of the course impacted your clinical practice? If yes, How?		
	No	33 (58.9)
	Yes	23 (41.1)
	**If yes, Stages of ** **Change**	
	Contemplation	7 (12.5)
	Determination	2 (3.6)
	Action	11 (9.6)
	Maintenance	3 (5.4)
4. Has the content of the course impacted your clinical practice due to the COVID-19 pandemic, the current social justice and equality movement, or the current healthcare policy debate? If yes, how?		
	No	40 (71.4)
	Yes	16 (28.6)
	**If yes, Stages ** **of Change**	
	Contemplation	8 (14.3)
	Determination	1 (1.8)
	Action	6 (10.7)
	Maintenance	1 (1.8)

### Impact on teaching and practice by stages of change

For impact of the course on teaching, 21 participants (37.5%) were not given a stage of change (SOC) because they reported that the course did not have an impact on their teaching and 33 participants were not given a SOC (58.9%) because they reported that the course did not have an impact on their practice ( Table 3). Of the 35 participants (62.5%) who reported that the course had an impact on their teaching, 12 participants (25.0%) were in the Contemplation stage (increased awareness/considering change), and 17 (30.4%) reported taking action (behavior change) due to the course. Of the 23 (41.1%) participants who reported that the course had an impact on their practice, 7 (12.5%) were in the Contemplation stage and 11 participants (9.6%)) reported taking action (behavior change) due to the course.

For the two questions about teaching and practice relative to the COVID-19 pandemic and the current social justice and equality movement, 26 participants (46.4%) reported that the course impacted their teaching and 16 participanrts (28.6%) reported that the course impacted their practice.

### Contemplation stage of change

The Contemplation stage of change is characterized by awareness of a problem and considering change. Examples of responses about the impact of the course on teaching that were assigned to the Contemplation stage of change are: “It has made me more aware of things I say, do and include in my courses and classrooms”, “Yes, I do think about bias all the time”, “Enhanced awareness” and “I have thought about it with respect to teaching, but am unsure how to make it actionable.” Examples of responses about the impact of the course on practice that were assigned to the Contemplation stage of change are: “try to be more aware of how cultural differences impact care decisions”, “yes, more aware of my implicit and explicit biases”, “I am more aware of how institutional racism might have impacted the patient's experience with the healthcare system”, and “As I am working with diverse patients I am aware of contributing to a culture of inclusion and respect for all backgrounds.”

### Action stage of change

We identified seven types of actions that participants implemented in teaching and provided illustrative quotes in Table 4: 1) include implicit bias education in curriculum, such as adding curriculum content related to micro- and macro-aggressions; 2) initiate discussions about implicit bias (participants reported feeling more comfortable to discuss biases with students with the information offered by the course); 3) teach students the impact of implicit bias on health disparities; 4) have more awareness of their own biases while mentoring trainees; 5) intentionally elevate the voices of individuals from underrepresented groups; 6) making diversity a priority in the recruitment process; and 7) become involved in training and service that promotes equity. Among the participants who reported taking action in their clinical practice due to the course, we identified five types of actions implemented with illustrative quotes in Table 4: 1) engage in reflective practice to think of patients in the broader sociocultural contexts; 2) provide empathetic listening when seeing patients and validating patients’ experiences and perspectives; 3) actively advocate for better access to care for patients of color; 4) improve the policies of practice by evaluating for implicit bias and seeking data; and 5) support antiracist social causes through participation and donations.

**Table 4.** Actions Taken that Participants Attributed to Implicit Bias Course Content.

EXAMPLES ACTIONS: TEACHING	
Themes	Illustrative Quotes
1. Add implicit bias education in curriculum	“Broadly shifted with increased awareness and have added curriculum on directly addressing micro and macro aggressions.”
2. Initiate discussions on implicit bias	“I'm more likely to bring this up as a topic for discussion with students, since I feel empowered to know there is literature/science out there to back up associations.”
3. Teach students the impact of implicit bias on health disparities	“It has been essential in helping students to understand why we shouldn't be surprised by the racial and social differences in outcomes.”
4. Be more aware of own biases in mentorship	“I am more aware of my own biases when providing additional resources and letters of recommendations for students.”
5. Elevate the voices of underrepresented groups	" I have been more cognizant of elevating the voices of individuals from underrepresented groups.”
6. Prioritize diversity in recruitment	“Recently hired a research assistant and care was taken to make diversity a priority.”
7. Involve in training and service that promote equity	“Yes, it has made me more passionate about this and I am now serving on our diversity/inclusion committee and helping with an anti-racism curriculum for our college.”
EXAMPLES ACTIONS: PRACTICE	
Themes	Illustrative Quotes
1. Engage in reflective practice	“It has altered my perspective on some patients' presentations. For instance, why they are seeking certain resources that others may not have. It has compelled me to take additional steps in some cases in an attempt to secure resources that I may not have previously.”
2. Provide empathetic listening	“I have been able to validate the experiences more of patients I have from marginalized groups.”
3. Advocate for patients of color	“I aim to dispel implicit bias on Patients of Color as I promote access to care during the COVID pandemic. I advocate more testing and treatment when indicated in this population since they are adversely affected by COVID.”
4. Improve the policies of practice	“Again, I think the overall increased trainings in implicit bias have given me more awareness and confidence in recognizing implicit bias in interpersonal interactions that I am involved with and observe. Also, when planning and implementing new practice policies, I think implicit bias is more part of the discussion.”
5. Support antiracist social causes	“I have participated in campus wide events designed to improve equity, increased donations targeted to be actively antiracist and continued to provide whatever support to trainees that I can while working remotely.”

### Implicit and explicit measures and course impact

We found no statistically significant correlation between implicit or explicit bias and impact on teaching or practice ( Table 5). We found a weak association between implicit race and explicit race measures indicating that these are related but distinct measures of race and gender biases. Implicit and explicit race and gender were associated suggesting that individuals who have bias in one area may hold implicit bias in other areas.

**Table 5.** Implicit and Explicit Bias and Course Impact on Teaching and Practice (N=56).

	Impact Teaching(r) ^a^	Impact Teaching Covid-19	Impact Practice	Impact Practice Covid-19	Implicit Race	Implicit Gender	Explicit Race	Explicit Gender (Career)	Explicit Gender (Family)
**Impact** **Teaching**	1.00								
**Impact ** **Teaching Covid**	-0.08	1.00							
**Impact** **Practice**	0.17	0.17	1.00						
**Impact ** **Practice Covid**	-0.04	0.07	0.51 **	1.00					
**Implicit Race**	0.14	-0.08	0.02	-0.01	1.00				
**Implicit Gender**	0.12	-0.01	0.05	0.19	0.26 **	1.00			
**Explicit Race**	0.09	-0.10	0.09	0.19	0.20 *	0.06	1.00		
**Explicit Gender ** **(Career)**	0.18	-0.17	0.08	0.06	0.03	0.18	0.09	1.00	
**Explicit Gender ** **(Family)**	0.19	0.02	-0.21	-0.16	-0.01	-0.13	-0.19 *	-0.41 **	1.00

a. Pearson Product Moment correlation (r) *p≤0.05 level **p ≤0.01 level

## Discussion

One year after completing a brief health equity/implicit bias course, we found that study participants experienced lasting effects that resulted in self-reported contemplation or awareness of bias and possible actions they might take (anticipatory change) and described actions they had taken due to the course (behavior change). To our knowledge this is the first study to measure lasting effects of brief, online health equity/implicit bias education after one year, implicit and explicit race and gender bias and participant reports on the impact of the course on their teaching and practice. Applying a Stages of Change Model ^24^ allowed us to assess responses that identify no change, contemplation of change, or new actions and behaviors taken directly attributed to the course. In the model ^24^, Contemplation is a pre-action stage. Participants in this study who demonstrated the Contemplation stage most often expressed a new awareness of health equity and bias in healthcare. Participants who demonstrated the Action stage described specific actions taken in their teaching and practice that they attributed to the course. Many participants attributed the course content to contemplating behavioral change or reported actual changes they had made in their teaching and practice due to the course over the span of one year. Our results suggest that it may take time for the effects of such education to lead to changes in behavior. This is an area that warrants further exploration.

Of particular interest are the actions (change in behavior) participants took over the course of the year that they directly attributed to the course. Few, if any, studies on effects of health equity/implicit bias education for academic clinicians have reported specific actions taken one year after a brief health equity/implicit bias course that learners directly attributed to course content. Much of the change reported in the literature is anticipatory. One study that evaluated training for residents and faculty found that six months later participants found the training increased commitment to addressing bias and institutional vigilance regarding implicit bias ^27^. Another study examined impact of health equity rounds on racism and implicit bias in patient care and found that the majority of participants reported that the health equity rounds would impact their future clinical practice ^28^. Our study is unique in that it finds that participants directly attributed course content to their behavior change one year following the course and described the real world actions they had taken. The variety of actions taken by participants suggest that there is potential for brief health equity/implicit bias education to impact specific activities in a wide range of primary care work settings found in academic medicine and other healthcare environments.

 Although our sample held race and gender biases, similar to bias in the general population and other clinicians ^5, 20, 29^, these biases were not associated with the impact of the course on Contemplation and Action. This suggests that regardless of the strength of clinician implicit or explicit bias, health equity/implicit bias education has the potential to increase awareness and motivate behavior change.

This study occurred during a significant period in U.S. history. Between the initial study which ended in late 2019 and the follow-up in late 2020, were the start of the COVID-19 pandemic, and the George Floyd and other police murders which reinvigorated social justice and Black Lives Matter movements. During the year 2020, existing societal inequalities were reported upon by the media and inescapable. While we were not able to separate the effects of the course, the effects of the pandemic and renewed focus on social inequities, we specifically asked participants whether “ *the content of the course impacted your teaching and/or mentoring”* and asked separately about impact of the course “ *due to the COVID-19 pandemic, the current social justice and equality movement, or current healthcare policy debate*.” The relevance of the course may have been magnified by social events occurring outside of academic medicine. Study participants were healthcare professionals who experienced the pandemic from both the perspective of health care provider, teacher, and citizen. We do not know whether or how these societal forces contributed to the impact of this specific health equity/implicit bias course or health equity/implicit bias education more broadly. Future research may provide answers to the influence of these forces on clinicians.

### Limitations

There are several limitations to our study. Our sample is a convenience sample, not a representative sample. Participants are academic primary care clinicians who were interested enough in the topic of implicit bias to give us one hour of their time in 2019 and another 30–45 minutes of their time one year later to participate in the current study. This sample is likely skewed toward clinicians who are not resistant to learning about racism, social determinants of health, and implicit bias in healthcare. In addition, it is likely that many participants were already well-versed in the topic of implicit bias in healthcare and as such may not be representative of the primary care clinician population. The questions we asked specifically referred to reflection on the impact of the course. We do not know how the events of the year 2020 in the U.S nor the global pandemic may have influenced participants’ responses. For those who reported no impact from the course, we were not able to assign a stage of change; it was not possible to determine whether there was no impact at all or no impact because they were already engaged in strategies to manage implicit bias in their setting. Although implicit and explicit bias measures were collected in the baseline study one year prior, research shows that these measures are fairly stable over time and are not easily mutable ^10^. Despite these limitations, this study found that brief health equity/implicit bias education can have lasting effects and, for some, motivate behavior change. Longitudinal evaluation of the impact of health equity/implicit bias education is recommended.

### Implications for education and practice

Brief health equity/implicit bias education is just one component of a systems-level comprehensive education plan, but it is an important one. Brief implicit bias education that is designed for academic clinicians who teach can, for many learners, motivate taking action to address bias and inequity in their teaching and practice environments.

## Ethics and consent

The University of Washington Human Subjects Institutional Review Board approved the study as minimal risk [Baseline study approval IRB # 00006978, Modification for follow up IRB #00008382, 11/13/2020]. Participants consented to the original study, and for the follow up study, participants who entered the online survey were first greeted with a new IRB approved consent form that explained the study. (Supplement 2.) To move forward into the study participants had to click an agree-to-participate button.

The University of Washington IRB “compliance is described in University of Washington (UW) Executive Order Number 24, and in UW’s Federal wide Assurance (FWA), UW and its IRBs are guided by the ethical principles in the Belmont Report. In addition, UW HSD and the UW IRBs draw upon a variety of ethical codes, such as the Declaration of Helsinki, the Council for International Organizations of Medical Sciences (CIOMS) and the International Council for Harmonization (ICH) when developing policies.”

## Data Availability

Due to ethical and security reasons and the need to protect data and participant privacy, we are not able to publicly share the data because we don’t have adequate consent from our participants. In the case of this study, even deidentified transcripts may be identifiable because of specific examples (e.g., specific actions participants reported) and contexts (setting specific information, etc.) participants shared, thus confidentiality may not be protected. Violation of confidentiality is of particular concern in this study due to the sensitive information that participants shared. For example, if a participant were identified, this could result in penalties. This study was approved by the University of Washington Institutional Review Board. For queries related to ethics of data access, contact Marya Kinsler from the UW IRB at 206-543-0471 or maryaj@uw.edu. We may consider requests for sharing de-identified data. In order to share the data on a case-by-case basis, we would need a description of purpose of use, credentials of those individuals who would work with the data, confirmation of ethics training of research team, requestor’s institutional IRB approval for the research, and UW IRB review and approval for secondary use of the data. In addition, we would need to take additional steps to remove any additional sensitive information. Data sharing requests may be granted only if the above conditions are met and the request is feasible. Please contact Drs. Sabin ( sabinja@uw.edu, or Bianca Frogner ( bfrogner@uw.edu) for data sharing requests. Lasting effects of brief health equity/implicit bias education for academic clinicians: From learning to action, https://doi.org/10.17605/OSF.IO/PGS8Q ^30^ This project contains the following underlying data: Underlying data file 1: dataset (Lasting Effects dataset) (restricted access) Underlying data file 2: dataset description (Lasting Effects dataset-codebook), Consent Form, Survey (unrestricted access)

## Software availability

Analysis can be done with open access software R: https://www.r-project.org/
