# Self-reported impacts one year after a brief health equity/implicit bias course for academic clinicians

**DOI:** 10.12688/mep.20799.2

**Published:** 2025-12-17

**Authors:** Janice Sabin, Grace Guenther, Kris Piu Kwan Ma, Bernadette York, Wendy Barrington, Bianca Frogner

**Affiliations:** 1 University of Washington School of Medicine, Seattle, Washington, USA; 2 University of Washington School of Nursing, Seattle, Washington, USA

**Keywords:** implicit bias education, lasting effects, implicit bias, explicit bias

## Abstract

**Purpose:**

The purpose of this study was to explore whether there were lasting effects of brief implicit bias education on clinicians’ teaching and practice one year after taking the course and whether implicit and explicit bias was associated with self-reported impact of the course.

**Method:**

This was a mixed-method study. We followed up with a sample of 119 academic clinicians who completed the baseline study December 2019. Recruitment for the current study was conducted between December 2020 and March 2021. Participants responded online to survey questions about whether the course had an impact on their teaching and practice. We categorized qualitative responses to these questions using Prochaska & DiClemente's Stages of Change Model of Behavior Change. Implicit and explicit race and gender bias data were collected at baseline.

**Results:**

Response rate was 47.1% (N=56). Participants were 64.3% female, 66.1% White, 67.9% were Medical Doctors (MD) and 82.1% work in an academic healthcare system. Overall, we found slight implicit pro-White bias (mean= 0.27, SD 0.45, p=<0.001) and male-career gender bias (mean= 0.33, SD 0.31, p=<0.001). Across all four questions 42 unique participants (75.0%) responded to at least one question reporting, “ *yes”, the course had an impact on their teaching/mentoring and or practice.* Thirty-five (62.5%) participants reported that the course had an impact on their teaching and 23 (41.4%) reported an impact on their clinical practice. Participants reported 35 instances of increased bias awareness and 47 instances of actions taken due to the course. Those who reported no impact of the course on teaching held no implicit race bias, while those who reported actions taken held moderate implicit Pro-White bias.

**Conclusions:**

This study found that the majority of study participants reported lasting effects of the course on their teaching and/or practice. Brief implicit bias education can impact clinicians’ teaching and practice.

## Introduction

Healthcare professionals’ implicit bias is one of many factors contributing to disparities in healthcare and health outcomes ^1, 2^. Implicit bias is defined as “attitudes or stereotypes that affect our understanding, decision-making and behavior without our even realizing it” ^3^. Similar to the general population, implicit bias exists among health professionals in the areas of race, gender, ethnicity, sexual orientation, age, weight, mental illness, and other areas ^1, 4– 6^. Healthcare professionals’ implicit bias contributes to poor clinician-patient communication, disparities in pain management, prescribing lipid-lowering medication for women, treatment of coronary heart disease, and other areas ^1, 7, 8^.

Implicit bias education for students, trainees and clinicians in practice has been integrated into some medical school curricula and healthcare system clinician trainings, but not all. There is no clear evidence of optimal implicit bias curricula content, or educational evaluation strategies ^9^ and no irrefutable evidence that implicit bias education impacts teaching and practice. The focus of most implicit bias education is not to eliminate the bias that is by its nature ubiquitous, hidden, and has proven to persist in the long term ^10^, but rather, to recognize how bias manifests and manage the impact of implicit attitudes and beliefs in teaching, practice and healthcare delivery ^11, 12^. At the University of Washington, authors of this study found that academic clinical faculty needed additional foundational health equity/implicit bias education to feel competent in teaching about implicit bias in healthcare. In 2017, we developed a brief, online course for academic clinicians titled, *Implicit Bias in the Clinical and Learning Environment,* to meet this need.

Evaluation of health equity/implicit bias education for clinical faculty who teach is in its early stages. Little is known about lasting effects of implicit bias education on clinicians’ behavior. This study was exploratory in nature. We did not know how implicit and explicit biases would affect reports of the impact of the course on teaching and practice in a one year follow up. Studies, including the baseline study from which we drew our sample ^13^, have found that brief online implicit bias education can increase bias awareness and intentions to change behavior ^13– 15^. In this study, we returned to a sample of primary care clinical faculty one year after they took a brief online course and used the method of personal reflection for participants to report on the impact of the course in their teaching and clinical practice over the past year. The aim of this study was to explore whether and how the course had an impact on clinicians’ teaching and practice during the one year following taking the course. Our research questions explored: 1. *Does health equity/implicit bias education have clinician self-reported lasting effects on teaching and practice, and if so, how?* and *2. Is clinician implicit and explicit bias associated with self-reported lasting effects of health equity/implicit bias education?*

## Methods

### Study design and sample

This mixed-method exploratory study returned to a sample of academic primary care clinicians who initially completed a survey and online health equity/implicit bias education, *Implicit Bias in the Clinical and Learning Environment* ^13^, between September 2019 and December 2019, which we refer to as our baseline study, course publicly available at [ https://depts.washington.edu/somalt/implicitbias-pi/story.html]. One year later, participants who completed the baseline study were invited to participate in the current follow up study to evaluate lasting effects of the course. The follow up study was conducted between December 2020 and March 2021. Our baseline sample consisted of 119 U.S. academic family, internal, and emergency medicine providers, nurse practitioners, and physician assistants recruited from all nine U.S. Census Divisions ^13^. Demographic and background information was collected in the baseline study, including information on personal and professional characteristics, and implicit and explicit race and gender bias measures. In the follow up survey, we updated participants’ work position and asked four reflection questions about if and how the course impacted their teaching and clinical practice over the past year. The University of Washington Human Subjects Institutional Review Board approved the study as minimal risk [Baseline study approval IRB # 00006978, Modification for follow up IRB #00008382, approved 11/13/2020].

**Implicit Bias Education:** Participants engaged in brief online health equity/implicit bias education as part of the baseline study. The course was developed by a team (including author JS), with expertise in medical education, adult teaching and learning theory, social determinants of health, implicit bias in healthcare, medical school clinician-administrators and academic clinicians who practice across a wide range of settings to serve as foundational information for academic clinicians who teach. The course was designed to be brief (35–40 minutes) so that it would not over burden busy clinicians. The course had three learning objectives: 1) define implicit bias and how it is manifested in health care, 2) recognize how implicit bias may be operating in the clinical setting and learning environment, and 3) apply strategies that can be used to minimize impact of implicit bias. Although the course was developed prior to publication of the Gonzalez *et al*., (2021) ^11^ framework of operationalizing implicit bias recognition and management (IBRM) education and the Sukhera and Watling (2018) ^16^, operationalization of a theoretical framework for implicit bias education, the course incorporated many of the features of these models such as creating a safe environment, content on the science of implicit bias, normalizing bias, evidence of bias in the learning environment and practice, and increasing awareness of implicit bias ^16^. Course content included: the history of racism in medicine, information about the social determinants of health, evidence of discrimination in healthcare, the science of implicit bias, and evidence about how implicit bias manifests in clinical care and the learning environment. Although skills practice was not part of the course, participants were provided with actionable strategies to mitigate the impact of bias in teaching and practice. Upon entering this follow up study, participants had the opportunity to revisit the course.

**Implicit Bias Measures:** In the baseline study we measured participant implicit race and gender bias using the standard Race Implicit Association Test (IAT) and Gender-Career IATs [available at: https://implicit.harvard.edu/implicit/] designed by scientists at Project Implicit using best practices for IAT design ^17, 18^. The IAT is a widely used, computer-based test of implicit social cognition that measures the relative strength between positive and negative associations toward one social group compared with another ^14^. The Black-White Race IAT asks test takers to sort and pair facial images of the target concept of race (faces of Black People and faces of White People) and words that represent “good” (e.g., glorious) or “bad” (e.g., yucky) as they appear on a computer screen. The Gender-Career IAT measures gender stereotypes using the target concepts of “male” (represented by traditionally male and female names, (e.g., Ben) versus “female” (e.g., Rebecca) and the concept of “career” represented by words associated with career (e.g., office) versus “family” represented by words associated with family (e.g., home). The difference in time taken to sort and pair these images and words as they rapidly appear on computer screen, measured in milliseconds, demonstrates the strength of their automatic association ^19^. The IAT and explicit measures were used as a one-time, baseline measure of bias to characterize the sample and was not used as an IAT with feedback intervention to increase awareness of participants’ personal bias. To explore the topic further, participants were given the Project Implicit web address [ https://implicit.harvard.edu/implicit/] which provides the public with an opportunity to take IATs with personal feedback. Implicit measures are a continuous measure ranging from -2.0, +2.0 with “0” signifying no bias.

**Explicit Bias Measures:** At baseline, we used standard explicit measures designed using best practices ^17^ that correspond with the Race IAT [available at: https://implicit.harvard.edu/implicit/] to measure respondents’ preference for Black People versus White People on a 7-point preference response scale ^20^. The explicit race measure question was: Which statement best describes you? *1*. *I strongly prefer Black People compared to White People; 2. I moderately prefer Black People compared to White People; 3. I slightly prefer Black People compared to White People; 4. I like Black People and White People equally; 5. I slightly prefer White People compared to Black People; 6. I moderately prefer White People compared to Black People; and 7. I strongly prefer White People compared to Black People*. For the Gender IAT, we used two separate explicit measures: one that asked about association of gender (male versus female) with career only, and one that asked about association of gender with “career” versus “family” only, using the same format and 7-point scale. Explicit measures were analyzed on a scale that ranges from -3.0, +3.0, with “0” signifying no bias.

**Open-ended, Reflective Questions:** Participants were asked four yes/no reflective questions and to provide a typed response to the following prompts: *1. Reflecting on this course, has the content of the course impacted your teaching and/or mentoring? If yes, how? 2. Has the content of the course impacted your teaching and/or mentoring due to the COVID-19 pandemic, the current social justice and equality movement, or the current healthcare policy debate? If yes, how? 3. Reflecting on this course, has the content of the course impacted your clinical practice? If yes, how? and 4. Has the content of the course impacted your clinical practice due to the COVID-19 pandemic, the current social justice and equality movement, or the current healthcare policy debate? If yes, how?* Supplement Table, Data repository [ **:** https://doi.org/10.17605/OSF.IO/PGS8Q ^21^]

**Qualitative Analysis:** We utilized a rapid practical thematic analysis approach for analysis of responses to the four reflective questions that aligns with the guide developed by Braun & Clarke ^22^, We used the Prochaska & DiClemente Stages of Change Model of Behavior Change guide ^23^ which has previously been used to categorize qualitative data from bias interventions, as our analytical framework ^24^. The stages of change categories are; Precontemplation (no intention to change), Contemplation (awareness of a problem,), Determination (preparing to change), Action (changed behavior), and Maintenance (maintaining new behavior) ^23^. Study team members (JS, GG, KM, WB, BWY, BF) independently coded a subset of reflective question responses according to the Stages of Change model. The study team then collaboratively reviewed and finalized codes through an iterative process. Once codes were finalized, one team member (GG) applied the codes to the remaining questions and responses. All team members collectively defined and finalized themes and sub-themes within each stage of the model. We analyzed responses using Microsoft Excel, with response by participant in separate cells and separate sheets. Stages of change were coded ordinally, with precontemplation scoring a 0, contemplation a 1, determination a 2, action a 3, and maintenance a 4.

**Quantitative Analysis:** Quantitative analysis consisted of descriptive statistics to characterize the sample and implicit and explicit bias scores and one-way between group analysis of variance (ANOVA) to explore differences in IAT scores, participant stages of change and impact of course. The IAT D score mean was derived using the standard IAT scoring algorithm ( 19, Greenwald, 2003). Quantitative analysis was conducted using Stata (StataCorp. 2019. Stata Statistical Software: Release 16. College Station, TX: StataCorp LLC.) ^25^ and IBM SPSS Statistics for Macintosh (version 26.0, Armonk, NY: IBM Corp) ^26^. [see software availability statement]

## Results

### Sample

***Participants***

Participants were 64.3% female, 66.1.0% White, 67.9% were Medical Doctors (MD), and 82.1% worked in an academic healthcare system ( Table 1). We compared those who responded to those who did not and found a few differences in characteristics of participants did and did not participate in the Y2 follow up study. Follow up study sample participants reported significantly less years in practice (16.0 years vs. 21.2 p=0.02), more worked in an academic healthcare system (82.1% vs. 63.2%%, p=0.04), and held stronger explicit gender bias (female/family mean= -1.07 vs. mean= -0.63, p=0.02). Supplement Table, Data repository [ **:** https://doi.org/10.17605/OSF.IO/PGS8Q ^21^]

**Table 1.** Study Sample Characteristics (N=56).

	N (%)
**Gender**	
Male	20 (35.7)
Female	36 (64.3)
**Age**	
30–39	23 (41.1)
40–49	17 (30.4)
50–59	8 (14.3)
60+	7 (12.5)
Prefer not to Answer	1 (1.8)
**Ethnicity**	
Hispanic/Latino	4 (7.1)
Not Hispanic/Latino	52 (92.9)
**Race**	
American Indian/Alaska Native	0 (0.0)
Asian	9 (16.1)
Black/African American	7 (12.5)
Native Hawaiian or Other Pacific Islander	0 (0.00)
White	37 (66.1)
Two or more races	1 (1.8)
Other	2 (3.57)
**Provider Type**	
Medical Doctor (MD)	38 (67.9)
Nurse Practitioner (NP)/Physician Assistant (PA)	9 (16.1)
Other	9 (16.1)
**Healthcare System**	
Academic Healthcare System	46 (82.1)
Community Healthcare System	9 (16.1)
Other	1 (1.8)
**Census Region**	
Northeast	9 (16.1)
Midwest	14 (25.0)
South	22 (39.3)
West	11 (19.6)
**Professional Experience**	**Mean (SD)**
Years in Clinical Practice Mean (SD)	16.0 (11.2)
Years in Current Position Mean (SD)	7.9 (8.3)
# Patients/Week Direct Clinical Contact Mean (SD)	21.4 (10.6)
**Implicit and Explicit Scores**	**Mean (SD) ^a^ | P-value ^b^**
Implicit Gender (IAT)	0.33 (0.31) | 0.000
Implicit Race (IAT)	0.27 (0.45) | 0.000
Explicit Race	0.11 (0.76) | 0.524
Explicit Gender – Career	0.76 (1.07) | 0.000
Explicit Gender – Family	-1.07 (0.95) | 0.000

a. For implicit and explicit measures, a positive score favors White, male with career, and female with family. A negative score favors Black, female with family. Interpretation of implicit and explicit measures: >-0.15 and > 0.15- little to no preference, > ±0.15 and 0.35 -slight preference, >±0.35 and 0.65 - moderate preference, > ±0.65 - strong preference b. T-test to determine whether mean differs from “0”

### Implicit and explicit measures

Overall, our sample held slight implicit pro-White bias (mean= 0.27, SD 0.45), and slight implicit gender bias associating males rather than females with the concept of “career” versus “family” (mean= 0.33, SD 0.31), ( Table 1). On explicit measures, we did not find evidence of explicit race bias (mean=0.11, SD 0.76). We found strong self-reported explicit bias associating “male” with “career” versus “family” (mean=0.76, SD 1.07), and female with “family” versus “career” (mean= -1.07, SD 0.95).

### Overall impact of the course

Across all four questions, 42 (75.0%) unique participants reported *“yes”, the course had an impact on their teaching/mentoring and/or practice* to at least one question, representing 35.3% of the original sample. Table 2. One year after taking the course, 35 participants (62.5%) reported that the content of the course had an impact on their teaching and 23 participants (41.4%) reported that the course had an impact on their clinical practice. ( Table 2). Those assigned to the Maintenance stage (n=4) reported that they were already engaged in strategies to manage implicit bias in their setting. Participants who responded “no” to a question were not assigned a stage of change (SOC). For the two questions about teaching and practice relative to the COVID-19 pandemic and the current social justice and equality movements, 26 participants (46.4%) reported that the course impacted their teaching and 16 participants (28.6%) reported that the course impacted their practice. Across all four questions, participants reported 35 instances of increased awareness of bias (Contemplation) and 47 self-reported instances of actions taken that they attributed to the content of the course.

**Table 2.** Impact of Course on Teaching and Practice and Stages of Change (N=56).

	N (%)
**“Yes” to at least 1 question:** the number of unique individuals who responded, *Yes,* the content of the course impacted my teaching/mentoring and/or practice.	42 (75.0%)
**1. Reflecting on this course, has the content of the course impacted your teaching and/or mentoring? If yes, How?**	
No	21 (37.5)
Yes	35 (62.5)
***If yes, Stages of Change***	
Contemplation	12 (34.3)
Determination	2 (5.7)
Action	17 (48.6)
Maintenance	4 (11.4)
**2. Has the content of the course impacted your teaching and/or mentoring due to the COVID-19 pandemic, the current social justice and equality movement, or the current healthcare policy debate? If yes, How?**	
No	30 (53.6)
Yes	26 (46.4)
***If yes, Stages of Change***	
Contemplation	8 (30.8)
Determination	2 (7.7)
Action	13 (50.0)
Maintenance	3 (11.5)
**3. Reflecting on this course, has the content of the course impacted your clinical practice? If yes, How?**	
No	33 (58.9)
Yes	23 (41.1)
***If yes, Stages of Change***	
Contemplation	7 (30.4)
Determination	2 (8.7)
Action	11 (47.8)
Maintenance	3 (13.0)
**4. Has the content of the course impacted your clinical practice due to the COVID-19 pandemic, the current social justice and equality movement, or the current healthcare policy debate? If yes, how?**	
No	40 (71.4)
Yes	16 (28.6)
***If yes, Stages of Change***	
Contemplation	8 (50.0)
Determination	1 (6.3)
Action	6 (37.5)
Maintenance	1 (6.3)

### Contemplation stage of change

The Contemplation stage of change is characterized by awareness of a problem ^23^. Examples of responses about the impact of the course on teaching that were assigned to the Contemplation stage of change are: “It has made me more aware of things I say, do and include in my courses and classrooms”, “Yes, I do think about bias all the time”, “Enhanced awareness” and “I have thought about it with respect to teaching, but am unsure how to make it actionable.” Examples of responses about the impact of the course on practice that were assigned to the Contemplation stage of change are: “try to be more aware of how cultural differences impact care decisions”, “yes, more aware of my implicit and explicit biases”, “I am more aware of how institutional racism might have impacted the patient's experience with the healthcare system”, and “As I am working with diverse patients I am aware of contributing to a culture of inclusion and respect for all backgrounds.”

### Action stage of change

We identified seven types of actions that participants implemented in teaching and provided illustrative quotes in Table 3.: 1) include implicit bias education in curriculum, such as adding curriculum content related to micro-and macro-aggressions; 2) initiate discussions about implicit bias (participants reported feeling more comfortable to discuss biases with students with the information offered by the course); 3) teach students the impact of implicit bias on health disparities; 4) more awareness of their own biases while engaging in mentoring trainees; 5) intentionally elevate the voices of individuals from underrepresented groups; 6) making diversity a priority in the recruitment process; and 7) become involved in training and service that promotes equity. Among the participants who reported taking action in their clinical practice due to the course, we identified five types of actions implemented with illustrative quotes in Table 3. 1) engage in reflective practice to think of patients in the broader sociocultural contexts; 2) provide empathetic listening when seeing patients and validating patients’ experiences and perspectives; 3) actively advocate for better access to care for patients of color; 4) improve the policies of practice by evaluating for implicit bias and seeking data; and 5) support antiracist social causes through participation and donations.

**Table 3.** Self-reported Actions Taken that Participants Attributed to Course Content.

EXAMPLES ACTIONS: TEACHING	
Themes	Illustrative Quotes
1. Add implicit bias education in curriculum	“Broadly shifted with increased awareness and have added curriculum on directly addressing micro and macro aggressions.”
2. Initiate discussions on implicit bias	“I'm more likely to bring this up as a topic for discussion with students, since I feel empowered to know there is literature/science out there to back up associations.”
3. Teach students the impact of implicit bias on health disparities	“It has been essential in helping students to understand why we shouldn't be surprised by the racial and social differences in outcomes.”
4. Be more aware of own biases when engaged in in mentorship	“I am more aware of my own biases when providing additional resources and letters of recommendations for students.”
5. Elevate the voices of underrepresented groups	" I have been more cognizant of elevating the voices of individuals from underrepresented groups.”
6. Prioritize diversity in recruitment	“Recently hired a research assistant and care was taken to make diversity a priority.”
7. Involve in training and service that promote equity	“Yes, it has made me more passionate about this and I am now serving on our diversity/inclusion committee and helping with an anti-racism curriculum for our college.”
EXAMPLES ACTIONS: PRACTICE	
Themes	Illustrative Quotes
1. Engage in reflective practice	“It has altered my perspective on some patients' presentations. For instance, why they are seeking certain resources that others may not have. It has compelled me to take additional steps in some cases in an attempt to secure resources that I may not have previously.”
2. Provide empathetic listening	“I have been able to validate the experiences more of patients I have from marginalized groups.”
3. Advocate for patients of color	“I aim to dispel implicit bias on Patients of Color as I promote access to care during the COVID pandemic. I advocate more testing and treatment when indicated in this population since they are adversely affected by COVID.”
4. Improve the policies of practice	“Again, I think the overall increased trainings in implicit bias have given me more awareness and confidence in recognizing implicit bias in interpersonal interactions that I am involved with and observe. Also, when planning and implementing new practice policies, I think implicit bias is more part of the discussion.”
5. Support antiracist social causes	“I have participated in campus wide events designed to improve equity, increased donations targeted to be actively antiracist and continued to provide whatever support to trainees that I can while working remotely.”

### Self-Reported impact of course on teaching and practice, implicit and explicit bias and stages of change

With the exception of implicit race bias and impact on teaching, there were no significant differences in stages of change, impact of course and implicit race and gender bias scores, with IAT scores for each stage being similar. Table 4. For impact of the course on teaching, there were significant differences in stages of change (p= 0.036) on impact of the course by participant implicit race bias. Participants who reported that the course had no impact on teaching had no implicit race bias (mean=0.12, SD 0.49, those who were in the Contemplation stage held slight implicit pro-White bias (mean=0.30, SD 0.34), and those who reported taking Action (behavior change) due to the course held moderate implicit pro-White bias (mean=0.44, SD 0.38). There were no differences in impact of the course on teaching and practice based on participants’ explicit race bias which showed little explicit race bias. Table 5. Explicit gender bias scores for all stages of change and for “no impact” revealed strong associations of male with career and female with family.

**Table 4.** Self-reported Impact of Course on Teaching and Practice, Implicit Bias Scores and Stages of Change (N=56).

TEACHING							
Implicit Race IAT				Implicit Gender IAT			
Stage of Change	N	Mean (SD) ^a^	P-value ^b^	Stage of Change	N	Mean (SD) ^a^	P-value
No	19	0.12 (0.49)	**0.036**	No	19	0.33 (0.32)	0.675
Contemplation	14	0.30 (0.34)		Contemplation	14	0.25 (0.34)	
Determination	2	0.80 (0.28)		Determination	2	0.52 (0.38)	
Action	17	0.44 (0.38)		Action	17	0.36 (0.28)	
Maintenance	4	-0.11 (0.56)		Maintenance	4	0.45 (0.37)	
PRACTICE							
Implicit Race IAT				Implicit Gender IAT			
No	33	0.25 (0.48)	0.988	No	33	0.31 (0.31)	0.903
Contemplation	7	0.32 (0.34)		Contemplation	7	0.37 (0.34)	
Determination	2	0.30 (0.30)		Determination	2	0.33 (0.44)	
Action	11	0.31 (0.48)		Action	11	0.40 (0.34)	
Maintenance	3	0.18 (0.61)		Maintenance	3	0.23 (0.22)	

a. For implicit measures, a positive score favors White, male with career. A negative score favors Black, female with family. Interpretation of implicit measures: >-0.15 and < 0.15- little to no preference, > ±0.15 and 0.35 -slight preference, >±0.35 and 0.65 - moderate preference, > ±0.65 - strong preference b. One-way between group analysis of variance (ANOVA) tests.

**Table 5.** Self-reported Impact of Course on Teaching and Practice, Explicit Bias Scores and Stages of Change (N=56).

TEACHING											
Explicit Race				Explicit Gender: Career				Explicit Gender: Family			
Stage of Change	N	Mean (SD) ^a^	P-value ^b^	Stage of Change	N	Mean (SD) ^a^	P-value ^b^	Stage of Change	N	Mean (SD) ^a^	P-value ^b^
No	19	0.00 (0.58)	0.505	No	19	0.63 (1.10)	0.211	No	19	-1.37 (0.90)	0.514
Contemplation	14	0.07 (1.07)		Contemplation	14	0.69 (1.10)		Contemplation	14	-1.00 (0.96)	
Determination	2	1.00 (0.00)		Determination	2	0.50 (0.71)		Determination	2	-0.50 (0.71)	
Action	17	0.19 (0.75)		Action	17	0.71 (0.85)		Action	17	-0.88 (0.93)	
Maintenance	4	0.00 (0.00)		Maintenance	4	2.00 (1.41)		Maintenance	4	-1.00 (1.41)	
PRACTICE											
Explicit Race				Explicit Gender: Career				Explicit Gender: Family			
No	33	0.06 (0.72)	0.242	No	33	0.66 (1.15)	0.6106	No	33	-0.94 (0.93)	0.473
Contemplation	7	0.29 (0.76)		Contemplation	7	1.14 (1.07)		Contemplation	7	-1.14 (1.07)	
Determination	2	-1.00 (0.00)		Determination	2	0.00 (1.41)		Determination	2	-0.50 (0.71)	
Action	11	0.27 (0.90)		Action	11	1.00 (0.77)		Action	11	-1.36 (1.03)	
Maintenance	3	0.33 (0.58)		Maintenance	3	0.67 (1.15)		Maintenance	3	-1.67 (0.58)	

a. For explicit measures, a positive score favors White, male with career. A negative score favors Black, female with family. Interpretation of explicit measures: >-0.15 and < 0.15- little to no preference, > ± 0.15 and 0.35 -slight preference, >± 0.35 and 0.65 - moderate preference, > ± 0.65 - strong preference. b. One-way between group analysis of variance (ANOVA) tests.

## Discussion

To our knowledge this is the first study to measure whether there were lasting effects of brief, online health equity/implicit bias education after one year, implicit and explicit race and gender biases and participant reports on the impact of the course on their teaching and practice. One year after completing a brief health equity/implicit bias course, we found that 42 (75.0%, and 35.3% of the original sample) answered *yes, the course had an impact*, to at least one of the four questions, with 62.5% of study responders reporting that the course had an impact on their teaching and 41.1% reporting an impact on their practice. Our study is unique in that participants directly attributed course content to their self-reported increased awareness of bias and reports of actions they took, suggesting that there is potential for brief health equity/implicit bias education to impact specific activities in a wide range of primary care work settings found in academic medicine and other healthcare environments.

Applying a Stages of Change Model ^23^ allowed us to assess responses that report no change, increased awareness (Contemplation), or new actions and behaviors taken directly attributed to the course. In the model ^23^, Contemplation is a pre-action stage. Participants in this study who demonstrated the Contemplation stage most often expressed a new awareness of health equity and bias in healthcare. Participants who demonstrated the Action stage described specific self-reported actions taken in their teaching and practice that they attributed to the course content. Our results suggest that it may take time for the effects of such education to manifest. For those who reported no impact, more ongoing exposure to implicit bias/health equity education may be needed, including direct skills building content into implicit bias education which has been shown to enhance the impact of implicit bias course content, (Gonzales *et al.*, 2022- Twelve Tips). In addition, there may be a portion of clinicians who just are not impacted by this type of education. This is an area that warrants further exploration.

Our sample held implicit and explicit biases, similar to bias in the general population and other clinicians ^5, 20, 27^. We found that strength of implicit race bias resulted in differences in impact of the course on teaching. Those who reported no impact of the course on their teaching held no implicit race bias, which may signify that was no impact because they had already incorporated the content of the course into their teaching and practice. Participants who reported taking action held moderate implicit Pro-White bias, the strongest race bias reported by stage of change. Perhaps those with greater implicit pro-White bias were motivated by the course content to take action in their teaching, compared to those who held no bias and reported no impact of the course. How implicit and explicit biases affect effectiveness and application of health equity/implicit bias education to teaching and practice is an area for further study.

Of particular interest are the self-reported actions (change in behavior) participants took over the course of the year that they directly attributed to the course. Few, if any, studies on effects of health equity/implicit bias education for academic clinicians have reported specific actions taken one year after a brief health equity/implicit bias course that learners directly attributed to course content. Much of the change reported in the literature is anticipatory. One study that evaluated training for residents and faculty found that six months later participants found the training increased commitment to addressing bias and institutional vigilance regarding implicit bias ^28^. Another study examined impact of health equity rounds on racism and implicit bias in patient care and found that the majority of participants reported that the health equity rounds would impact their future clinical practice ^29^.

This study occurred during a significant period in U.S. history. Between the initial study which ended in 2019 and the follow-up in late 2020, were the start of the COVID-19 pandemic, and the George Floyd and other police murders which reinvigorated social justice and Black Lives Matter movements. Study participants were healthcare professionals who experienced the pandemic from both the perspective of health care provider, teacher, and citizen. During the year 2020, existing societal inequalities were reported upon by the media and inescapable. While we were not able to separate the effects of the course, the effects of the pandemic and renewed focus on social inequities, we specifically asked participants whether “ *the content of the course impacted your teaching and/or mentoring”* and asked separately about impact of the course “ *due to the COVID-19 pandemic, the current social justice and equality movement, or current healthcare policy debate*.” The relevance of the course may have been magnified by social events occurring outside of academic medicine. We do not know whether or how these societal forces contributed to the impact of this specific health equity/implicit bias course or health equity/implicit bias education more broadly. Future research may provide answers to the influence of these forces on clinician education.

### Limitations

There are several limitations to our study. Our sample is a convenience sample, not a representative sample. We had a 47.1% response rate, almost one half of our original sample. It is remarkable that participants gave us their time during the height of the COVID-19 pandemic when demands on primary care providers’ time was at a maximum. Participants are academic primary care clinicians who were interested enough in the topic of implicit bias to give us one hour of their time in 2019 and another 30–45 minutes of their time one year later to participate in the current study. This sample is likely skewed toward clinicians who are not resistant to learning about racism, social determinants of health, and implicit bias in healthcare. In addition, it is likely that many participants were already well-versed in the topic of implicit bias in healthcare and as such may not be representative of the primary care clinician population. Reliability of the IAT has been questioned; however, we used best practices in design and implementation of the IAT in research and explicit measures that adjust for specific critiques of the IAT ^17, 30^. For example, we used IATs in a single-session, which is considered adequately reliable for reporting group differences but not for individual diagnostic purposes, we used standard 7-Block IAT construction, target concepts and exemplar stimuli were developed according to best practices and our analysis used the standard IAT D score algorithm ^18^. For the qualitative analysis, coders who were trained in identifying stages of change using a practical thematic analysis strategy were aware of the study’s purpose which may have had an impact on coding. While 75.0% (n=42) of the Y2 study participants reported lasting effects on teaching and/or practice for at least one question, this represents 35.3% of the original sample from which study participants were drawn. The impact of the course was determined through self-report, which is not considered as reliable as direct objective observation. We do not know how the events of the year 2020 may have influenced participants’ responses. Although implicit and explicit bias measures were collected in the baseline study one year prior, research shows that these measures are fairly stable over time and are not easily mutable ^10^. Despite these limitations, this study found that brief health equity/implicit bias education can have lasting effects and, for some, motivate self-reported behavior change. Longitudinal evaluation of the impact of health equity/implicit bias education is recommended.

### Implications for education and practice

Brief health equity/implicit bias education is just one component of a systems-level comprehensive education plan, but it is an important one. Brief implicit bias education that is designed for academic clinicians who teach can, for many learners, increase awareness of bias and motivate taking action to address bias and inequity in their teaching and practice environments.

## Ethics and consent

The University of Washington Human Subjects Institutional Review Board approved the study as minimal risk [Baseline study approval IRB # 00006978, Modification for follow up IRB #00008382, 11/13/2020]. Participants consented to the original study, and for the follow up study, participants who entered the online survey were first greeted with a new IRB approved consent form that explained the study. (Supplement 2.) To move forward into the study participants had to click an agree-to-participate button.

The University of Washington IRB “compliance is described in University of Washington (UW) Executive Order Number 24, and in UW’s Federal wide Assurance (FWA), UW and its IRBs are guided by the ethical principles in the Belmont Report. In addition, UW HSD and the UW IRBs draw upon a variety of ethical codes, such as the Declaration of Helsinki, the Council for International Organizations of Medical Sciences (CIOMS) and the International Council for Harmonization (ICH) when developing policies.”

## Data Availability

Due to ethical and security reasons and the need to protect data and participant privacy, we are not able to publicly share the data because we don’t have adequate consent from our participants. In the case of this study, even deidentified transcripts may be identifiable because of specific examples (e.g., specific actions participants reported) and contexts (setting specific information, etc.) participants shared, thus confidentiality may not be protected. Violation of confidentiality is of particular concern in this study due to the sensitive information that participants shared. For example, if a participant were identified, this could result in penalties. This study was approved by the University of Washington Institutional Review Board. For queries related to ethics of data access, contact Marya Kinsler from the UW IRB at 206-543-0471 or maryaj@uw.edu. We may consider requests for sharing de-identified data. In order to share the data on a case-by-case basis, we would need a description of purpose of use, credentials of those individuals who would work with the data, confirmation of ethics training of research team, requestor’s institutional IRB approval for the research, and UW IRB review and approval for secondary use of the data. In addition, we would need to take additional steps to remove any additional sensitive information. Data sharing requests may be granted only if the above conditions are met and the request is feasible. Please contact Drs. Sabin ( sabinja@uw.edu, or Bianca Frogner ( bfrogner@uw.edu) for data sharing requests. Lasting effects of brief health equity/implicit bias education for academic clinicians: From learning to action, https://doi.org/10.17605/OSF.IO/PGS8Q ^21^ This project contains the following underlying data: Underlying data file 1: dataset (Lasting Effects dataset) (restricted access) Underlying data file 2: dataset description (Lasting Effects dataset-codebook), Consent Form, Survey (unrestricted access) Underlying data file 3: Supplement Table: Sample Non-responders versus Responders Comparisons (N=114)

## Software availability

Analysis can be done with open access software R: https://www.r-project.org/
