# Supplementary material for: Self-reported impacts one year after a brief health equity/implicit bias course for academic clinicians
Source: MedEdPublish (2016). 2025 Dec 17;15:32. Originally published 2025 Jul 11. [Version 2] doi: 10.12688/mep.20799.2 (PMC12759272; doi:10.12688/mep.20799.2)
Supplement: Peer review history

# Reviewer report: Reviewer response for version 2

**DOI:** 10.21956/mep.22958.r44891

**Published:** 2026-01-02

**Authors:** Yannick Eller, Mehrdad Heravi

**Affiliations:** 1 University of Dundee, Dundee, Scotland, UK; 2 Isfahan University of Medical Sciences, Isfahan, Isfahan Province, Iran

I have read the authors’ responses and the revised manuscript. The authors have addressed the principal concerns we raised. The title has been revised to clarify outcomes were self-reported, the methods section now clarifies the sampling and coding process for the qualitative data and the staged coding approach, the statistical analyses have been reworked and the limitations have been expanded to explicitly acknowledge the constraints. No further comments. Thank you to the authors and the editorial team for the thorough and constructive revisions.

Have any limitations of the research been acknowledged?

Yes

Is the work clearly and accurately presented and does it cite the current literature?

Partly

If applicable, is the statistical analysis and its interpretation appropriate?

Partly

Are all the source data underlying the results available to ensure full reproducibility?

Partly

Is the study design appropriate and is the work technically sound?

Partly

Are the conclusions drawn adequately supported by the results?

No

Are sufficient details of methods and analysis provided to allow replication by others?

No

Reviewer Expertise:

Faculty development; competency-based training and assessment; simulation-based education and patient-safety improvement; interdisciplinary clinical education with application to oncology and imaging. This is an intentionally concise, high-level summary - please consult online data or prior reviews for detailed listings.

We confirm that we have read this submission and believe that we have an appropriate level of expertise to confirm that it is of an acceptable scientific standard.

# Reviewer report: Reviewer response for version 1

**DOI:** 10.21956/mep.22263.r43316

**Published:** 2025-10-30

**Authors:** Yannick Eller, Mehrdad Heravi

**Affiliations:** 1 University of Dundee, Dundee, Scotland, UK; 2 Isfahan University of Medical Sciences, Isfahan, Isfahan Province, Iran

The current title’s phrase ‘Lasting effects’ risks implying objectively measured, durable behaviour change; because this manuscript reports participant self-reports rather than direct observation or administrative outcomes, I suggest a gentler phrasing that aligns title and evidence—for example, ‘Self-reported impacts one year after a brief implicit-bias/health-equity course for academic clinicians’ or ‘Participant-reported impacts one year following a brief implicit-bias course’. Either option preserves the emphasis on follow-up while making the evidence base explicit and avoiding overstated causal language.

 The term "brief education" in the title may not fully capture the nature and scope of the intervention described in the article. Furthermore, the phrase “health equity” is used broadly, but the study principally measures race- and gender-related implicit attitudes; the title should either reflect that narrower focus or the authors should justify the broader EDI claim. Revising the title with these considerations could enhance reader comprehension and engagement.

 The summary is clearly written and provides a concise overview of the study’s scope and findings. However, the Abstract and Introduction currently overstate the strength of the evidence: the manuscript uses baseline IATs and follow-up self-reports, which are best described as Kirkpatrick Level-1 (awareness/reaction) outcomes. It would strengthen the rationale to explicitly state that this study aims not only to explore additional impacts beyond awareness, such as behavioral change, but also to acknowledge the limits of self-report follow-up designs for demonstrating such change. Furthermore, regarding the second research question on the association between clinician implicit and explicit bias and lasting educational effects, the current study’s results appear to align more closely with the first research question; the manuscript should therefore clarify how the quantitative and qualitative strands were intended to be integrated to address the second question.

 The methods section is well articulated and clearly describes how data were gathered and analyzed, providing transparency and rigor in the study’s approach. That said, the manuscript’s repeated use of “multi-method” is not currently justified: the authors should either describe a prespecified mixed-methods design (and how integration occurred) or relabel the study as a quantitative baseline with a qualitative follow-up. The methodology appears to be more directly aligned with addressing the first research question regarding the overall effects of the education program. It is less clear how the methods specifically support the investigation of the second question related to the association between clinicians' implicit and explicit bias and lasting educational effects. Please clarify sampling logic for the follow-up, who coded the qualitative responses, how many responses were double-coded, intercoder reliability (or how coder agreement was handled), and how saturation was judged. Clarifying this alignment in the methods would strengthen the coherence and focus of the study design.

 The results are clearly presented, with well-organized tables and text that effectively summarize the key findings without over-interpretation. The clarity of the presentation allows readers to readily understand the outcomes of the study, supporting transparency and comprehension.

 The discussion is well supported by the study’s results, effectively integrating findings with relevant context. Still, the manuscript would benefit from a clearer limitation statement that distinguishes self-reported actions from objectively observed behaviour change (Kirkpatrick Level 3) and that proposes concrete Level 3/4 follow-up designs (for example, matched-control or stepped-wedge trials, objective audits, or patient-level outcome tracking). A particularly positive aspect is the acknowledgment of the COVID-19 pandemic and local social activities as potential influences on the study outcomes, which demonstrates thoughtful consideration of external factors that may impact implicit bias education and its effects.

 The take-home message is concise and effectively summarizes the key findings and significance of the study. I would, however, soften language that implies verified “lasting effects” and reframe conclusions to reflect participant reports of impact rather than definitive evidence of behaviour change. Additionally, the references are well curated, relevant, and support the arguments presented throughout the article.

 Evaluation

 Quality of science: X_lower 50% __upper 50% __top 25% __top 10%

 Impact/significance/applicability: __lower 50% X_upper 50% __top 25% __top 10%

 Originality/novelty: __lower 50% X_upper 50% __top 25% __top 10%

 Absolute score: 58% - promising, but requires substantive reframing, additional methodological transparency, and reanalysis.

 Verdict: Revise & Resubmit.”

Have any limitations of the research been acknowledged?

Yes

Is the work clearly and accurately presented and does it cite the current literature?

Partly

If applicable, is the statistical analysis and its interpretation appropriate?

Partly

Are all the source data underlying the results available to ensure full reproducibility?

Partly

Is the study design appropriate and is the work technically sound?

Partly

Are the conclusions drawn adequately supported by the results?

No

Are sufficient details of methods and analysis provided to allow replication by others?

No

Reviewer Expertise:

Faculty development; competency-based training and assessment; simulation-based education and patient-safety improvement; interdisciplinary clinical education with application to oncology and imaging. This is an intentionally concise, high-level summary - please consult online data or prior reviews for detailed listings.

We confirm that we have read this submission and believe that we have an appropriate level of expertise to confirm that it is of an acceptable scientific standard, however we have significant reservations, as outlined above.

# Response

**Published:** 2025-12-03

**Authors:** Janice Sabin

**Affiliations:** Biomedical Informatics and Medical Education, University of Washington, Seattle, WA, USA

**Thank you for your comments. Please see the comments to reviewers 1, 2, and 3 in addition to these.** **Revision: We have changed the title to: Self-reported impacts one year after a brief health equity/implicit bias course for academic clinicians **   Either option preserves the emphasis on follow-up while making the evidence base explicit and avoiding overstated causal language.

  The term "brief education" in the title may not fully capture the nature and scope of the intervention described in the article. Furthermore, the phrase “health equity” is used broadly, but the study principally measures race- and gender-related implicit attitudes; the title should either reflect that narrower focus or the authors should justify the broader EDI claim. Revising the title with these considerations could enhance reader comprehension and engagement.

 The summary is clearly written and provides a concise overview of the study’s scope and findings. However, the Abstract and Introduction currently overstate the strength of the evidence: the manuscript uses baseline IATs and follow-up self-reports, which are best described as Kirkpatrick Level-1 (awareness/reaction) outcomes.  It would strengthen the rationale to explicitly state that this study aims not only to explore additional impacts beyond awareness, such as behavioral change, but also to acknowledge the limits of self-report follow-up designs for demonstrating such change. Furthermore, regarding the second research question on the association between clinician implicit and explicit bias and lasting educational effects, the current study’s results appear to align more closely with the first research question; the manuscript should therefore clarify how the quantitative and qualitative strands were intended to be integrated to address the second question.

**Revision: We added to the limitations- The impact of the course was determined through self-report, which is not considered as reliable as direct objective observation. (p19)**

   The methods section is well articulated and clearly describes how data were gathered and analyzed, providing transparency and rigor in the study’s approach. That said, the manuscript’s repeated use of “multi-method” is not currently justified: the authors should either describe a prespecified mixed-methods design (and how integration occurred) or relabel the study as a quantitative baseline with a qualitative follow-up. The methodology appears to be more directly aligned with addressing the first research question regarding the overall effects of the education program. It is less clear how the methods specifically support the investigation of the second question related to the association between clinicians' implicit and explicit bias and lasting educational effects. Please clarify sampling logic for the follow-up, who coded the qualitative responses, how many responses were double-coded, intercoder reliability (or how coder agreement was handled), and how saturation was judged. Clarifying this alignment in the methods would strengthen the coherence and focus of the study design.

**Revision:** **This mixed-method, exploratory study returned to a sample of academic primary care clinicians who completed a survey and online health equity/implicit bias education, *Implicit Bias in the Clinical and Learning Environment*** **^14^** **, between September 2019 and December 2019, which we refer to as our baseline study, course publicly available at [** **https://depts.washington.edu/somalt/implicitbias-pi/story.html** **].  (p5)** **Participants who completed the baseline study were invited to participate in the current follow up study to evaluate lasting effects of the course. The follow up study was conducted between December 2020 and March 2021. (p5)** **Clarification of coding** **Revision: We utilized a rapid practical thematic analysis approach for analysis of  responses to the four reflective questions that aligns with the guide developed by Braun & Clarke** **[GG1]** **[GG2]** **.(new CITE), We used the Prochaska & DiClemente Stages of Change Model of Behavior Change guide ^24 ^which has previously been used to categorize qualitative data from bias interventions, as our analytical framework ^25 ^. The stages of change categories are; Precontemplation (no intention to change), Contemplation (awareness of a problem,), Determination (preparing to change), Action (changed behavior), and Maintenance (maintaining new behavior) ^24 ^. Study team members (JS, GG, KM, WB, BWY, BF) independently coded a subset of reflective question responses according to the Stages of Change model. The study team then collaboratively reviewed and finalized codes through an iterative process. Once codes were finalized, one team member (GG) applied the codes to the remaining questions and responses. All team members collectively defined and finalized themes and sub-themes within each stage of the model. We analyzed responses using Microsoft Excel, with response by participant in separate cells and separate sheets. Stages of change were coded ordinally, with precontemplation scoring a 0, contemplation a 1, determination a 2, action a 3, and maintenance a 4. (p7)**

# Reviewer report: Reviewer response for version 1

**DOI:** 10.21956/mep.22263.r42790

**Published:** 2025-09-04

**Authors:** Cristina M Gonzales

**Affiliations:** 1 New York University Grossman School of Medicine, New York, New York, USA

I appreciate the opportunity to review this important work on a construct that is of great interest to me. I commend the authors on seeking to investigate the effect of their intervention beyond the immediate post-intervention stage. Overall, I am pleased to see the authors striving to link implicit bias instruction to educationally and clinically meaningful outcomes. I do have some significant concerns that I detail below, and offer suggestions in a collegial spirit.

 Abstract:

 Results: The findings are all self-reported. In the abstract (and throughout the manuscript as detailed below) the authors over-state their findings. I am unable to see how the numbers add up to 63.8% of participants having taken at least one action, even just accounting for the sample size of responders (N=56). I calculate 50% but and even then, how did the authors account for the same participant taking an action in both their teaching and their clinical practice? Is this double counting accounted for? Regardless of what the accurate number should be, once that is addressed I suggest the authors revise the last sentence in the results so that it reads…(self-reported actual behavior change)…given no actual behavior change was objectively observed.

 Conclusion: Please add self-reported to this text as well. The final sentence is an overstatement of the findings. There is no evidence that any actions improved either teaching or practice. The participants who reported taking action reported applying what they learned to their teaching and practice (and even then, it would be a stretch to say that given we do not know the content of what the training).

 Keywords: separate out implicit bias and explicit bias

 Introduction:

 For the research question- what was answered was lasting self-reported effects. Suggest revising the research question to reflect what was answered.

 In the section Implicit Bias Education: the first sentence is confusing as written.

 The IBRM framework was not developed or described by Sukhera and Watling in 2018. That framework uses the term recognition to mean awareness, and gives guidance on raising awareness in self and others about implicit bias, and guidance on integrating it implicit bias instruction into health professions education. However, there is no skills-based component to it. IBRM actually emerged from Gonzalez’s lab (the exact term of implicit bias recognition and management—IBRM—has been written in grant application from that lab since 2011). In 2020 Gonzalez partnered with Sukhera and Watling to overlay IBRM onto transformative learning theory, and in 2021 Gonzalez partnered with Lypson and Sukhera to write a 12 tips paper operationalizing the IBRM framework into actual teaching practice. Please use the latter reference (2021) and if space allows the former and revise the sentence to accurately reflect the development of the IBRM framework.

 Methods:

 For the IAT, the D scores reported by Project Implicit are not interpreted in the same way as Cohen’s d scores. This will be confusing to any reader who is familiar with D score reporting with IATs. Please revise to reflect the D scores (which are continuous variables reported as categories of neutral, moderate, and strong preferences for ease of interpretation) with the appropriate cutoffs (+/- 0.15, +/- 0.35, and +/- 0.65, respectively).

 Results:

 As above, I do not understand how the authors reached the 63.8% value and the potential for double counting. The values in the paragraph under table 2 do not add up. Suggest increased clarity of reporting in the calculations.

 Further, more than a third and more than half of participants did not report an impact of the course on their teaching or practice, respectively. These large values require the authors to soften the interpretations and conclusions, as they are currently overstated.

 In Actions stage of change: #4 is not an action. Increased awareness without skill development can actually have negative consequences such as avoidance, interracial anxiety, and more. If they have awareness of their biases while mentoring trainees, what action are they actually taking? This also gets back to the problem of claiming improvement in teaching and practice. Suggest moving #4 to the contemplation stage and recalculating the numbers in the results section.

 Discussion:

 The first sentence overstates the findings. Only some participants experienced lasting effects (and for practice, actually less than half did).

 At the end of the first paragraph, although the results may suggest that it may take time for the effects of such education to lead to changes in behaviors, the fact that more than one third and more than one half of participants did not experience changes in behavior in teaching or practice, respectively may also suggest the following: some participants may need more instruction, alternative methods, or may simply be unreachable due to the nature of the construct. One can't assume more time would have given them the opportunity to change, especially for those who said it had no impact.

 Limitations: The reported long-lasting impacts of the intervention affected a subset of respondents who were a subset of the original sample who participated in the intervention. The authors need to engage more fully with that limitation. If the authors are unable to account for double counting in their calculations (i.e. the same participant took action in both their teaching and practice as a result of the intervention), then this needs to be discussed in the limitations.

Have any limitations of the research been acknowledged?

Partly

Is the work clearly and accurately presented and does it cite the current literature?

Partly

If applicable, is the statistical analysis and its interpretation appropriate?

No

Are all the source data underlying the results available to ensure full reproducibility?

Yes

Is the study design appropriate and is the work technically sound?

Yes

Are the conclusions drawn adequately supported by the results?

Partly

Are sufficient details of methods and analysis provided to allow replication by others?

Partly

Reviewer Expertise:

My lab developed the framework of Implicit Bias Recognition and Management and we develop and evaluate skills-based, behavioral interventions to address the impact of implicit bias on clinical encounters.

I confirm that I have read this submission and believe that I have an appropriate level of expertise to confirm that it is of an acceptable scientific standard, however I have significant reservations, as outlined above.

# Response

**Published:** 2025-12-03

**Authors:** Janice Sabin

**Affiliations:** Biomedical Informatics and Medical Education, University of Washington, Seattle, WA, USA

**Thank you for your comments. We clarified the impact and numbers, identifying unique participant responses.** **Revision: Across all four questions, 42 (75.0%) unique participants reported *“yes”, the course had an impact on their teaching/mentoring and/or practice* to at least one question, representing 35.3% of the original sample. Table 2.** **One year after taking the course, 35 participants (62.5%) reported that the content of the course had an impact on their teaching and 23 participants (41.4%) reported that the course had an impact on their clinical practice. (Table 2). Those assigned to the Maintenance stage (n=4) reported that they were already engaged in strategies to manage implicit bias in their setting.** **(p10)** **Revision: We added to the limitations section:** **For the qualitative analysis, coders who were trained in identifying stages of change using a practical thematic analysis strategy were aware of the study’s purpose which may have had an impact on coding.** **While 75.0% (n=42) of the Y2 study participants reported lasting effects on teaching and/or practice for at least one question, this represents 35.3% of the original sample from which study participants were drawn.** **(p19)** **Revision: We adjusted our description to read: self-reported awareness and actions throughout.** **We added to the limitations: The impact of the course was determined through self-report, which is not considered as reliable as direct objective observation. (p19)** Regardless of what the accurate number should be, once that is addressed I suggest the authors revise the last sentence in the results so that it reads…(self-reported actual behavior change)…given no actual behavior change was objectively observed.

 Conclusion: Please add self-reported to this text as well. The final sentence is an overstatement of the findings. There is no evidence that any actions improved either teaching or practice. The participants who reported taking action reported applying what they learned to their teaching and practice (and even then, it would be a stretch to say that given we do not know the content of what the training).

 Keywords: separate out implicit bias and explicit bias: **Done**

 Introduction:

 For the research question- what was answered was lasting self-reported effects. Suggest revising the research question to reflect what was answered. **Revised research questions:** **Our research questions were 1. *Does health equity/implicit bias education have clinician’s self-reported lasting effects on teaching and practice, and if so, how?* and *2. Is clinician implicit and explicit bias associated with self-reported lasting effects of health equity/implicit bias education? *(p5)**

   In the section Implicit Bias Education: the first sentence is confusing as written. **Revision: Implicit Bias Education** **Participants engaged in brief online health equity/implicit bias education as part of the baseline study. (p5)**

 The IBRM framework was not developed or described by Sukhera and Watling in 2018. That framework uses the term recognition to mean awareness and gives guidance on raising awareness in self and others about implicit bias, and guidance on integrating it implicit bias instruction into health professions education. However, there is no skills-based component to it. IBRM actually emerged from Gonzalez’s lab (the exact term of implicit bias recognition and management—IBRM—has been written in grant application from that lab since 2011). In 2020 Gonzalez partnered with Sukhera and Watling to overlay IBRM onto transformative learning theory, and in 2021 Gonzalez partnered with Lypson and Sukhera to write a 12 tips paper operationalizing the IBRM framework into actual teaching practice. Please use the latter reference (2021) and if space allows the former and revise the sentence to accurately reflect the development of the IBRM framework. **Revision:** **Although the course was developed prior to publication of the Gonzalez et al., (2021) (add Cite #11) framework of operationalizing implicit bias recognition and management (IBRM) education and the Sukhera and Watling (2018) (add CITE #16), operationalization of a theoretical framework for implicit bias education, the course incorporated many of the features of these models such as creating a safe environment, content on the science of implicit bias, normalizing bias, evidence of bias in the learning environment and practice, and increasing awareness of implicit bias** **^16^** **. Course content included: the history of racism in medicine, information about the social determinants of health, evidence of discrimination in healthcare, the science of implicit bias, and evidence about how implicit bias manifests in clinical care and the learning environment. Although skills practice was not part of the course, participants were provided with actionable strategies to mitigate the impact of bias in teaching and practice (p 6)**

 Methods:

 For the IAT, the D scores reported by Project Implicit are not interpreted in the same way as Cohen’s d scores. This will be confusing to any reader who is familiar with D score reporting with IATs. Please revise to reflect the D scores (which are continuous variables reported as categories of neutral, moderate, and strong preferences for ease of interpretation) with the appropriate cutoffs (+/- 0.15, +/- 0.35, and +/- 0.65, respectively). **To avoid confusion we removed the statistic Cohen’s *d* and revised our analysis and IAT interpretation: We added IAT and explicit measures mean, SD, p value based upon differing from “0” and the following interpretation was added to the tables:** **Revision:** **For implicit and explicit measures, a positive score favors White, and male with career, and female with family. A negative score favors Black, female with family. Interpretation of implicit and explicit measures: >-0.15 and < 0.15- little to no preference, > ±0.15 and 0.35 -slight preference, >±0.35 and 0.65 - moderate preference, > ±0.65 - strong preference** **.  ** Results:

 As above, I do not understand how the authors reached the 63.8% value and the potential for double counting. The values in the paragraph under table 2 do not add up. Suggest increased clarity of reporting in the calculations.

 Further, more than a third and more than half of participants did not report an impact of the course on their teaching or practice, respectively. These large values require the authors to soften the interpretations and conclusions, as they are currently overstated. **Revision: Across all four questions, 42 (75.0%) unique participants reported that “yes”, the course had an impact on their teaching/mentoring and/or practice to at least one question, representing 35.3% of the original sample. Table 2.** **One year after taking the course, 35 participants (62.5%) reported that the content of the course had an impact on their teaching/mentoring and 23 participants (41.4%) reported that the course had an impact on their clinical practice. (Table 2). Those assigned to the Maintenance stage (n=4) reported that they were already engaged in strategies to manage implicit bias in their setting. Participants who responded “no” to a question were not assigned a stage of change (SOC) because they reported that the course did not have an impact on their teaching/mentoring and practice. For the two questions about teaching and practice relative to the COVID-19 pandemic and the current social justice and equality movements, 26 participants (46.4%) reported that the course impacted their teaching and 16 participants (28.6%) reported that the course impacted their practice.** **Across all four questions, participants reported** **34 instances of increased awareness of bias (Contemplation) and 47 self-reported actions taken that they attributed to the content of the course. (p10)** In Actions stage of change: #4 is not an action. Increased awareness without skill development can actually have negative consequences such as avoidance, interracial anxiety, and more. If they have awareness of their biases while mentoring trainees, what action are they actually taking? This also gets back to the problem of claiming improvement in teaching and practice. Suggest moving #4 to the contemplation stage and recalculating the numbers in the results section.

   # **4 “I am more aware of my own biases when providing additional resources and letters of recommendations for students.”** **Participants’ responses were very straightforward for the most part. In our qualitative analysis meetings coders discussed this particular response and decided that it was an action, not merely a general increased awareness but applying increased awareness while engaging in the action of “providing” resources and letters of recommendation. We again reviewed this response and stand by our coding of action on this response. This was a direct uptake of the course content, which presented evidence of gender bias in letters of recommendation, how to identify the bias, and its impact for women in medicine. We clarified our analysis in Table 3. by explaining in the theme that participants were engaging in mentoring.** **Revision: Table 3, theme # 4:** **Aware of own biases while engaging in mentorship activities** Discussion:

 The first sentence overstates the findings. Only some participants experienced lasting effects (and for practice, actually less than half did). **We reviewed responses to the four questions and found that 42 unique participants answered yes to at least one question, thus 75% of participants were impacted by the course.** **Revision: To our knowledge this is the first study to measure whether there were lasting effects** **of brief, online health equity/implicit bias education after one year, implicit and explicit race and gender bias** **es** **and participant reports on the impact of the course on their teaching and practice.** **One year after completing a brief health equity/implicit bias course, we found that 42 (75.0%, and 35.3% of the original sample) answered *yes,*** ***the course had*** ***an impact*** **, to at least one of the four questions, with** **62.5% of** **study responders reporting** **that the course had an impact on their teaching and 41.1% report** **ing** **an impact on their practice.** **Our study is unique in that participants directly attributed course content to their self-reported increased awareness of bias and reports of actions they took, suggesting that there is potential for brief health equity/implicit bias education to impact specific activities in a wide range of primary care work settings found in academic medicine and other healthcare environments. (p17)**

 At the end of the first paragraph, although the results may suggest that it may take time for the effects of such education to lead to changes in behaviors, the fact that more than one third and more than one half of participants did not experience changes in behavior in teaching or practice, respectively may also suggest the following: some participants may need more instruction, alternative methods, or may simply be unreachable due to the nature of the construct. One can't assume more time would have given them the opportunity to change, especially for those who said it had no impact. **Revision: For those who reported no impact, more ongoing exposure to implicit bias/health equity education may be needed, including direct skills building content into implicit bias education which has been shown to enhance the impact of implicit bias course content, (Gonzales et. al., 2022- Twelve Tips). In addition, there may be a portion of clinicians who just are not impacted by this type of education.This is an area that warrants further exploration**. **(p17)**

 Limitations: The reported long-lasting impacts of the intervention affected a subset of respondents who were a subset of the original sample who participated in the intervention. The authors need to engage more fully with that limitation. If the authors are unable to account for double counting in their calculations (i.e. the same participant took action in both their teaching and practice as a result of the intervention), then this needs to be discussed in the limitations. **We clarified the numbers. See above**

# Reviewer report: Reviewer response for version 1

**DOI:** 10.21956/mep.22263.r42787

**Published:** 2025-09-02

**Authors:** Dipesh P Gopal

**Affiliations:** 1 Queen Mary University of London, London, England, UK

Firstly I welcome the authors' tireless efforts to change the norm. The write-up is comprehensive with clear mention of the methods, sample and limitations.

 IAT testing is used before and after but the non-significant nature of the after effects of the teaching in not in the abstract. "We found no statistically significant correlation between implicit or explicit bias and impact on teaching or practice ( Table 5). We found a weak association between implicit race and explicit race measures indicating that these are related but distinct measures of race and gender biases. Implicit and explicit race and gender were associated suggesting that individuals who have bias in one area may hold implicit bias in other areas."

 As we know self-reported change does not necessarily reflect change. Would a vignette study have been helpful here?

 The conclusions are misleading: "There were lasting effects of implicit bias education on participants’ teaching and practice. Brief implicit bias education moved clinicians toward taking action to improve their teaching and practice. "

 This study further adds to the growing literature that IAT testing / bias training does not significantly change views / minds / actions and this should be acknowledged in the study. There is even some research that bias training worsens existing biases.

Have any limitations of the research been acknowledged?

Yes

Is the work clearly and accurately presented and does it cite the current literature?

Yes

If applicable, is the statistical analysis and its interpretation appropriate?

Yes

Are all the source data underlying the results available to ensure full reproducibility?

Yes

Is the study design appropriate and is the work technically sound?

Partly

Are the conclusions drawn adequately supported by the results?

Partly

Are sufficient details of methods and analysis provided to allow replication by others?

Yes

Reviewer Expertise:

Inequalities, have reviewed the literature on bias and published a well cited review. I am familiar with bias testing and the limits of IAT testing.

I confirm that I have read this submission and believe that I have an appropriate level of expertise to confirm that it is of an acceptable scientific standard, however I have significant reservations, as outlined above.

# Response

**Published:** 2025-12-03

**Authors:** Janice Sabin

**Affiliations:** Biomedical Informatics and Medical Education, University of Washington, Seattle, WA, USA

**Thank you for your comments. We clarified that the implicit and explicit measures were one-time baseline measures to characterize the sample.** **Revision:** **The IAT and explicit measures were used as a one-time, baseline measure of bias to characterize the sample and was not used as an IAT with feedback intervention to increase awareness of participants’ personal bias. (p6)** "We found no statistically significant correlation between implicit or explicit bias and impact on teaching or practice ( Table 5). We found a weak association between implicit race and explicit race measures indicating that these are related but distinct measures of race and gender biases. Implicit and explicit race and gender were associated suggesting that individuals who have bias in one area may hold implicit bias in other areas."

**We changed our analysis from Pearson Product Moment correlation tests to a one-way between group analysis of variance (ANOVA) tests to better understand course impact on teaching and practice in relation to implicit and explicit bias scores and stages of change categories. (see Tables 4, 5)**   As we know self-reported change does not necessarily reflect change. Would a vignette study have been helpful here? **A vignette study was beyond the scope of this study but would be useful in future studies.**

**For all mentions of awareness of bias and actions taken we added that they were self-reported throughout the manuscript and added this to the limitations.**

   The conclusions are misleading: "There were lasting effects of implicit bias education on participants’ teaching and practice. Brief implicit bias education moved clinicians toward taking action to improve their teaching and practice. "

**Revision:** **Abstract** **Conclusions:** **This study found that the majority of study participants reported lasting effects of the course on their teaching and/or practice. Brief implicit bias education can impact clinicians’ teaching and practice. (p5)**   This study further adds to the growing literature that IAT testing / bias training does not significantly change views / minds / actions and this should be acknowledged in the study. There is even some research that bias training worsens existing biases. **We addressed the topic by adding to this sentence. There is no clear evidence of optimal implicit bias curricula content, or educational evaluation strategies and no irrefutable evidence that implicit bias education impacts teaching and practice** ^9^ ^.^ **(p 4)**

# Reviewer report: Reviewer response for version 1

**DOI:** 10.21956/mep.22263.r42537

**Published:** 2025-08-19

**Authors:** Jeffrey Stone

**Affiliations:** 1 Northern Arizona University, Flagstaff, Arizona, USA

This paper reports the results of a study designed to examine the longer term effects of implicit bias education and training among providers over a 1 year period.  It is an important study that will make a contribution to the literature on implicit bias interventions in health care.  I would like to see the research published at some point.  

 However, I am  concerned that the current paper over interprets the results in a way that could be misleading.  I offer the following observations and suggestions to clarify the results and improve the impact of the research.  

 1.  The "lasting effects" in the title are very limited to a small subsample of those who completed the follow-up.  For example, only 47% (57 of 119) completed the follow up, and of those, only 65% (35 of the 57) said the intervention had an impact on them.  Thus, the claims of lasting effects are for only 29% of those exposed to the intervention, a critical limitation that is not adequately acknowledged.  Moreover, who were the non-respondents?  No information is provided about those who did not complete the follow-up (e.g., the demographics of the respondents suggest attrition was predominantly White males). We have to consider the possibility that the intervention was ineffective on providers who tend to show high bias and are the most resistant to learning about it.  It should be possible to address the non-response rate by reporting the demographics of those who did not respond (this appears possible from the way they collected the pre-intervention data).   It might be possible to address these concerns by, for example, reporting the pre-intervention D-score on the race and gender IATs on the full sample of 119, and comparing it to the subsample of 57 who completed the follow-up.   

 2.  The coding of the stages of change were not conducted by coders who were unaware of the study purpose.  I worry that the coding may show more change than objective or naive coders would find.  It might be difficult to rule this out without training naive coders and having them replicate the results.  But the paper could acknowledge this potential limitation and its remedy (which other investigators can test if the data are made available).  

 3.    it could be misleading to interpret the zero-order correlation between the bias and change scores as evidence for the effectiveness of the intervention.  The zero correlation could reflect a restriction in range or the unreliability of one or both measures, for example.  The poor reliability of the IAT is well documented, and the paper does not report information about the reliability of the explicit measures used in this study.  The paper should acknowledge and/or address this possible explanation.   

 But the introduction did not say anything about whether bias scores measured before the intervention should  correlate with the outcomes.  Assuming the measures are valid and reliable, should there be no correlation, as the paper suggests, or should there be a positive correlation, which would indicate that the most biased providers were the most motivated by the intervention to contemplate or enact change?  Isnt that the goal of the intervention?  To clarify, the paper should provide more explanation of the expected relationship between the pre-intervention measures of bias and the outcomes.

Have any limitations of the research been acknowledged?

Partly

Is the work clearly and accurately presented and does it cite the current literature?

Partly

If applicable, is the statistical analysis and its interpretation appropriate?

No

Are all the source data underlying the results available to ensure full reproducibility?

Partly

Is the study design appropriate and is the work technically sound?

Partly

Are the conclusions drawn adequately supported by the results?

No

Are sufficient details of methods and analysis provided to allow replication by others?

No

Reviewer Expertise:

Implicit bias in health care

I confirm that I have read this submission and believe that I have an appropriate level of expertise to confirm that it is of an acceptable scientific standard, however I have significant reservations, as outlined above.

# Response

**Published:** 2025-12-03

**Authors:** Janice Sabin

**Affiliations:** Biomedical Informatics and Medical Education, University of Washington, Seattle, WA, USA

**Thank you for your comments. We have changed the title to be more descriptive of our findings.** **The revised title is *: *Self-reported impacts one year after a brief health equity/implicit bias course for academic clinicians** **We also clarify the impact of the course shown in Table 2.** **We added the following information:** **Revision: Across all four questions 42 (75.0%) unique participants responded reported that “yes”, the course had an impact on their teaching/mentoring and/or practice to at least one question, representing 35.3% of the original sample. (p10)** **Revision: We added to the limitations section: While 75.0% (n=42) of the Y2 study participants reported that yes, the course had an impact on teaching/practice, to at least one question, this represents 35.3% of the original sample from which study participants were drawn. (p19)** Moreover, who were the non-respondents?  No information is provided about those who did not complete the follow-up (e.g., the demographics of the respondents suggest attrition was predominantly White males). We have to consider the possibility that the intervention was ineffective on providers who tend to show high bias and are the most resistant to learning about it. **See Supplement Table:** https://doi.org/10.17605/OSF.IO/PGS8Q ^30^ It should be possible to address the non-response rate by reporting the demographics of those who did not respond (this appears possible from the way they collected the pre-intervention data). **Revision: Yes, we can address this question. We created a Supplemental Table that compares those from the original sample who did not respond in Year 2. to those who completed the Year 2 survey and report on the table on page 8.  See Supplement Table:** https://doi.org/10.17605/OSF.IO/PGS8Q ^30^ It might be possible to address these concerns by, for example, reporting the pre-intervention D-score on the race and gender IATs on the full sample of 119, and comparing it to the subsample of 57 who completed the follow-up. **See Supplement Table:** https://doi.org/10.17605/OSF.IO/PGS8Q ^30^ **We added to the text:** **We compared those who responded to those who did not and found a few differences in characteristics of participants did and did not participate in the Y2 follow up study. Follow up study sample participants reported significantly less years in practice (16.0 years vs. 21.2 p=0.02), more worked in an academic healthcare system (82.1% vs. 63.2%%, p=0.04), and held stronger explicit gender bias (female/family mean= -1.07 vs. mean= -0.63, p=0.02). Supplement Table, Data repository [** **:** **https://doi.org/10.17605/OSF.IO/PGS8Q** **^30^** **]** **(p8)** 2. The coding of the stages of change were not conducted by coders who were unaware of the study purpose.  I worry that the coding may show more change than objective or naive coders would find.  It might be difficult to rule this out without training naive coders and having them replicate the results.  But the paper could acknowledge this potential limitation and its remedy (which other investigators can test if the data are made available). **Revision:** **Limitations: For the qualitative analysis, coders who were trained in identifying stages of change using a practical thematic analysis strategy were aware of the study’s purpose which may have had an impact on coding. (p18)**

 3.    it could be misleading to interpret the zero-order correlation between the bias and change scores as evidence for the effectiveness of the intervention.  The zero correlation could reflect a restriction in range or the unreliability of one or both measures, for example.  The poor reliability of the IAT is well documented, and the paper does not report information about the reliability of the explicit measures used in this study.  The paper should acknowledge and/or address this possible explanation. **(see below)** **Revision:** **Limitations: Reliability of the IAT has been questioned, however, we used best practices in design and implementation of the IAT in research that adjust for specific critiques of the IAT.  (Greenwald, 2022, Nosek, 2007) For example, we used 2 IATs in a single-session, which is considered adequately reliable for reporting group differences, we used standard 7-Block IAT construction, target concepts and stimuli were developed according to best practices and analysis used the standard IAT D scoring algorithm.  (p18)**

**Revision: Explicit Measures: We used standard explicit measures designed using best practices outlined in Greenwald, et al., 2022) that correspond with the Race IAT.  (p6)** [ https://implicit.harvard.edu/implicit/]

   But the introduction did not say anything about whether bias scores measured before the intervention should correlate with the outcomes.  Assuming the measures are valid and reliable, should there be no correlation, as the paper suggests, or should there be a positive correlation, which would indicate that the most biased providers were the most motivated by the intervention to contemplate or enact change? **We changed our analysis from Pearson Product Moment correlation tests to a one-way between group analysis of variance (ANOVA) tests to better understand course impact on teaching and practice in relation to implicit and explicit bias scores and stages of change categories. We examined stages of change and differences of participant implicit and explicit bias scores. (see Tables 4, 5)** **Revision: With the exception of the impact of implicit race bias on teaching, there were no significant differences by stages of change in impact of course and implicit race and gender bias scores, with IAT scores for each stage being similar and ranging from slight to moderate bias.** **Table 4** **. For impact of course on teaching, there were significant differences in stages of change (p= 0.036) on impact of the course by participant implicit race bias. Participants who reported that the course had no impact on teaching had no implicit race bias (mean=0.12, SD 0.49), those who were in the Contemplation stage held slight implicit pro-White bias (mean=0.30, SD 0.34), and those who reported taking Action (behavior change) due to the course held moderate implicit pro-White bias (mean=0.44, SD 0.38).** **There were no differences in impact of the course on teaching and practice based on participants’ explicit race biases which showed little to slight explicit race bias. Table 5.  Explicit gender bias scores for all stages of change and for no impact revealed strong associations of male with career and female with family. Table 5. (p17)** Isn't that the goal of the intervention?  To clarify, the paper should provide more explanation of the expected relationship between the pre-intervention measures of bias and the outcomes. **Revision:** **This study was exploratory in nature.** **We did not know how implicit and explicit biases would affect reports of the impact of the course on teaching and practice in a one year follow up.** **Studies, including the baseline study from which we drew our sample, (add Cite # 14)) have found that brief online implicit bias education can increase bias awareness and intentions to change behavior** **^13^** **^–^** **^15^** **. In this study, we returned to a sample of primary care clinical faculty one year after they took a brief online course and used the method of personal reflection for participants to report on the impact of the course in their teaching and clinical practice over the past year. The aim of this study was to explore whether and how the course had an impact on clinicians’ teaching and practice during the one year following taking the course. (p5)**
